# Stimulation of hepatocarcinogenesis by activated cholangiocytes via Il17a/f1 pathway in *kras* transgenic zebrafish model

**DOI:** 10.1038/s41598-020-80621-6

**Published:** 2021-01-14

**Authors:** Mohamed Helal, Chuan Yan, Zhiyuan Gong

**Affiliations:** 1 Department of Biological Sciences, National University of Singapore, Singapore, Singapore; 2 Marine Pollution Lab, Marine Environment Division, National Institute of Oceanography and Fisheries, Alexandria, Egypt

**Keywords:** Cancer, Genetics, Molecular biology

## Abstract

It has been well known that tumor progression is dependent on secreted factors not only from tumor cells but also from other surrounding non-tumor cells. In the current study, we investigated the role of cholangiocytes during hepatocarcinogenesis following induction of oncogenic *kras*^*V12*^ expression in hepatocytes using an inducible transgenic zebrafish model. Upon induction of carcinogenesis in hepatocytes, a progressive cell proliferation in cholangiocytes was observed. The proliferative response in cholangiocytes was induced by enhanced lipogenesis and bile acids secretion from hepatocytes through activation of Sphingosine 1 phosphate receptor 2 (S1pr2), a known cholangiocyte receptor involving in cholangiocyte proliferation. Enhancement and inhibition of S1pr2 could accelerate or inhibit cholangiocyte proliferation and hepatocarcinogenesis respectively. Gene expression analysis of hepatocytes and cholangiocytes showed that cholangiocytes stimulated carcinogenesis in hepatocytes via an inflammatory cytokine, Il17a/f1, which activated its receptor (Il17ra1a) on hepatocytes and enhanced hepatocarcinogenesis via an ERK dependent pathway. Thus, the enhancing effect of cholangiocytes on hepatocarcinogenesis is likely via an inflammatory loop.

## Introduction

Liver cancer is one of the most prevalent cancers and it is ranked the fifth most common cancer and the second most cause of cancer related mortality with a dismal five-year survival rate of 10%^1–3^. Hepatocellular carcinoma (HCC), which is developed from hepatocytes, is the primary liver cancer. Several etiological factors contribute to HCC development, including hepatitis viral infection, cirrhosis, nonalcoholic fatty liver disease, environmental toxin, alcoholism, etc.^4–6^. Now several gene mutations causing increased susceptibility for liver cancers have also been identified such as *TERT* (telomerase reverse transcriptase), *TP53*, *CTNNB1* (β-catenin), etc.^7^.These studies highlights the importance of both genetic and environmental factors in promoting liver cancers.

In the liver, hepatocytes and cholangiocytes are two main cell types. Cholangiocytes are small, heterogeneous and dynamic group of epithelial cells lining the biliary ducts in the liver and have important absorptive and secretory functions^8–13^. Cholangiocytes are generally quiescent and are activated only under certain liver inflammatory or pathological conditions^8–12,14^. Several molecular pathways have been demonstrated to be involved in cholangiocyte proliferation^15–18^ such as Tgfb^19^, Notch^20,21^ and Hedgehog signalling pathways^12^. Various receptors are also known to control cholangiocyte proliferation and determine their response upon injury^13,22^. Among these receptors, Sphingosine 1 phosphate receptor 2 (S1pr2) appears to be a strong mediator of cholangiocyte proliferation in pathological and oncogenic conditions of cholangiocytes^23,24^.

In our laboratory, we have previously developed several transgenic zebrafish models for HCC by inducible expression of a selected oncogene in hepatocytes, such as *kras*^*V12*^^25,26^, *Myc*^27^, *xmrk*^28^ and *tgf1b*^29^. HCC are induced robustly from all of these transgenic zebrafish and they show resemblance to human liver cancer at both histological and molecular levels^30,31^. These inducible transgenic models allow us to control transgenic oncogene expression at desired time points to initiate tumorigenesis, thus providing valuable tools for investigation of tumour initiation, which may be difficult to achieve in human clinical studies and other animal models.

The importance of tumor microenvironment for tumor initiation and progression has been increasingly recognized^32,33^. In liver tumorigenesis, it is interesting to investigate the interaction between hepatocytes and surrounding cellular/non-cellular components. In zebrafish, several previous studies have shown that non-tumour cells can contribute to HCC progression through different secreted mediators, e.g. hepatic stellate cells via serotonin secretion^34^, immune cells neutrophils and macrophages via transforming growth factor beta (Tgfb)^35^ and cortisol^36^. As cholangiocytes are the second largest cell population in the liver, it is tempting to investigate the roles of cholangiocytes to development of HCC. In particular, cholangiocytes have been shown to have a strong connection to several liver diseases such as liver fibrosis^11^, cholestatic liver injury^37^, primary sclerosing cholangitis^38^ and also ethionine-induced HCC^39^. Hence, in this study, we attempted to examine the role of cholangiocytes during HCC initiation and progression using *kras*^*V12*^ transgenic zebrafish model we earlier established^26^. Our experiments demonstrated that cholangiocytes play an important role in promoting HCC development through an inflammatory pathway.

## Results

### Increase of cholangiocytes upon induction of oncogenic *kras*^*V12*^ expression in hepatocytes of *kras*^*V12*^ transgenic zebrafish larvae

**Figure 1:**
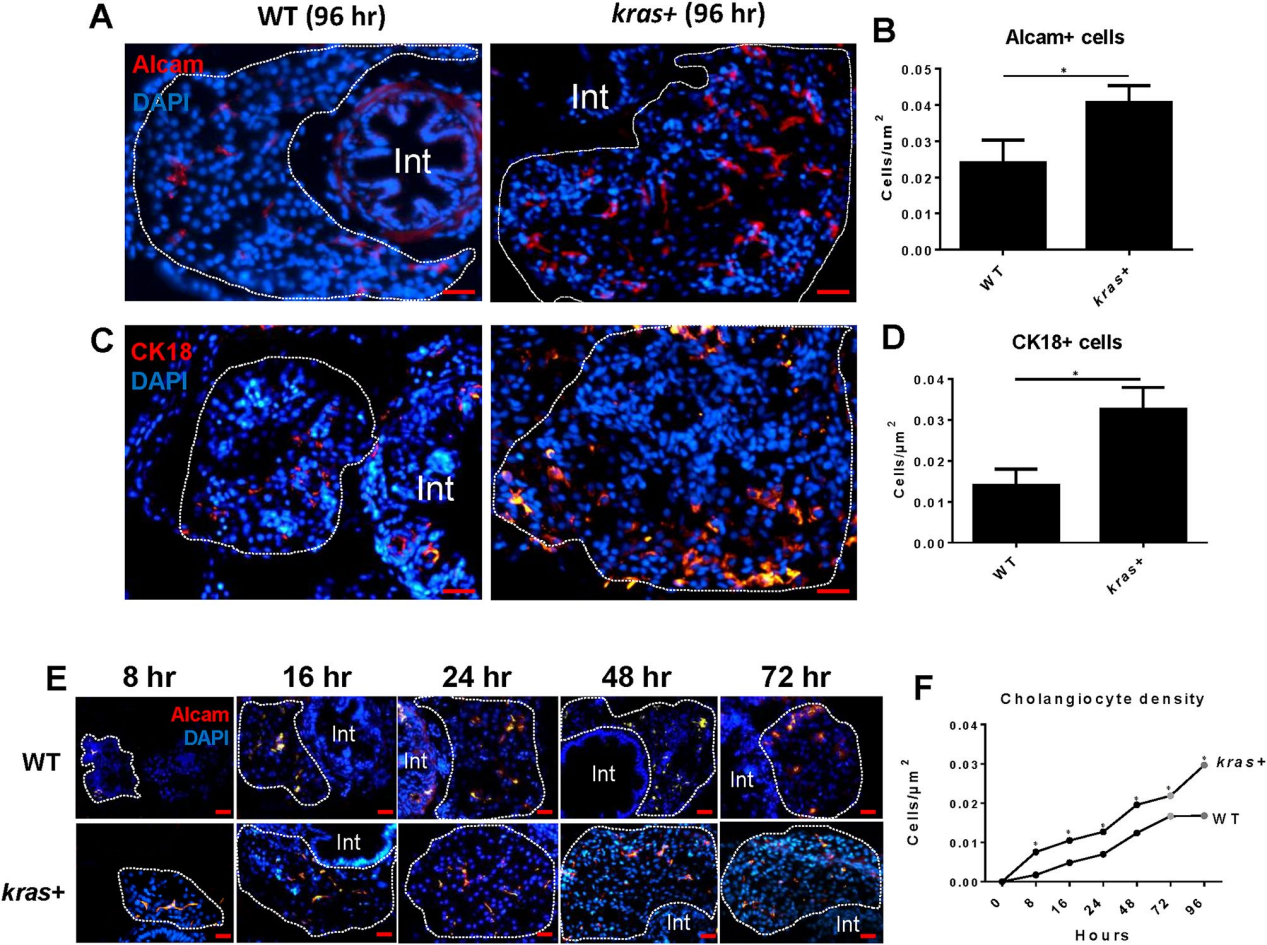
Increase of cholangiocytes upon induction of oncogenic *kras*^*V12*^ expression in hepatocytes of *kras*+ transgenic zebrafish larvae. Cholangiocytes were determined by using two different molecular markers: Alcam and Cytokeratin 18 (CK18). 3-dpf *kras*+ and WT zebrafish larvae were treated with 20 µg/ml Dox till 7 dpf. Samples were collected at different time points after Dox induction for immunohistochemistry. All liver sections were counter stained with DAPI. (**A**–**D**) Representative images of Alcam antibody (**A**) and Cytokeratin 18 (Ck18) (**C**) and quantification of cholangiocyte density (**B**,**D**) in *kras* + and WT larvae at 96 h following Dox induction. (**E**,**F**) Time course of increase of cholangiocytes from 8 to 96 h following Dox induction. Representative images of Alcam staining of *kras*+ and WT larvae from 8 to 72 h are shown in (**E**) and 96 h in (**A**). Quantification of cholangiocyte density in *kras*+ and WT larvae is shown in (**F**). N = 10 at each time point. Scale bar: 20 μm. Statistical significance: *P˂0.05.

To visualize the response of cholangiocytes upon *kras*^*V12*^ induction in hepatocytes. 3-dpf (day postfertilization) *kras*^*V12*^ transgenic (shorted as *kras* + in this report) larvae were induced by doxycycline (Dox) for 5 days to initiate hepatocarcinogenesis. Two specific cholangiocyte markers, Alcam and Cytokeratin 18 (Ck18), were used for identifying cholangiocytes in the liver sections. As shown in Fig. 1A–D, both Alcam and Ck18 stained cholangiocytes showed significant increases in the *kras* + group in comparison with the wildtype (WT) control group upon induction of oncogenic *kras*^*V12*^ expression by Dox. Cholangiocyte density was determined following Dox induction from 8 to 96 h. A shown in Fig. 1E,F, Alcam staining revealed significant increases in cholangiocyte density in the *kras* + group compared to the WT group at all time points and a significant increase was observed as early as 8 h after the initiation of Dox treatment.

### Acceleration of tumor growth via S1pr2 induction

Several cholangiocyte receptors have been reported to control cholangiocyte proliferation under different physiological and pathological conditions^8,9,11,40^. These receptors include secretin receptor (Sctr)^41^, estrogen receptor (Esr), insulin receptor (Insra and Insrb), glucagon-like-peptide receptor 2 (Glpr2), nerve growth factor receptor (Ngfr) and sphingosine 1 phosphate receptor 2 (S1pr2)^40,42–45^, etc. Expression of these cholangiocyte receptor genes in cholangiocytes and hepatocytes of WT and *kras* + adult zebrafish after Dox induction were analyzed by RT-qPCR. As shown in Supplementary Fig. S1, majority of these receptor genes showed little changes in expression in cholangiocytes and hepatocytes following oncogenic *kras*^*V12*^ expression in hepatocytes, but *s1pr2* expression had striking increases in both hepatocytes (28 fold) and cholangiocytes (> 120 fold). This is reminiscence of several previous reports that *s1pr2* is highly expressed in cholangiocarcinoma and bile duct cell diseases^43,46^. Thus, in view of high *s1pr2* expression in cholangiocytes and its correlation with cholangiocarcinoma and cholestatic liver disease, we chose *s1pr2* as a promising candidate receptor to study its role during HCC progression in our zebrafish model.

**Figure 2:**
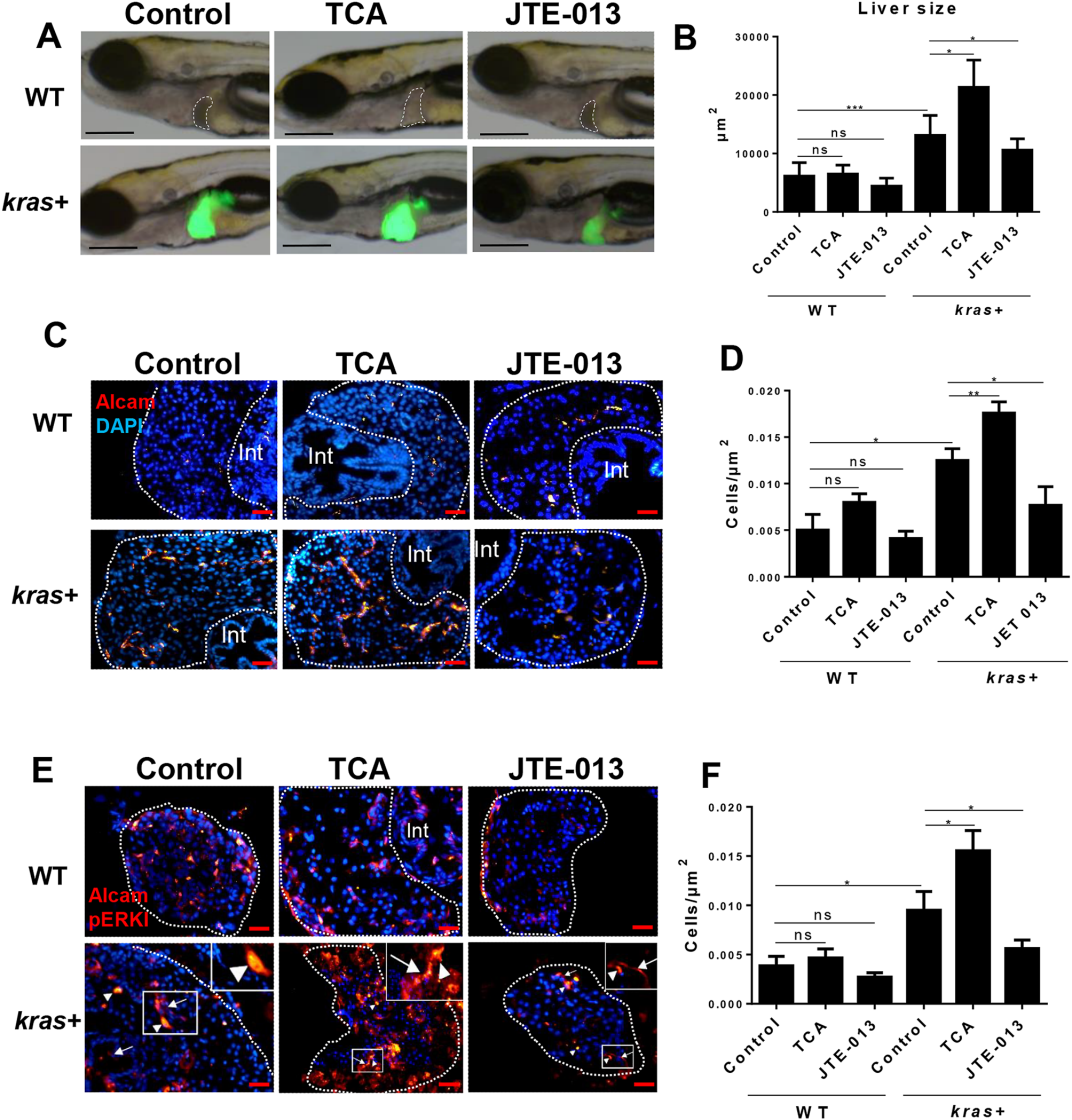
Effect of S1pr2 activation and inhibition on liver size, cholangiocyte density, and downstream marker pERK in *kras*+ and WT zebrafish larvae. 3-dpf *kras*+ and WT zebrafish larvae were treated with either TCA or JTE-013 along with 20 µg/mL Dox till 8 dpf. Samples were collected for immunohistochemistry and all liver sections were counter-stained with DAPI. (**A**) Representative images for liver size after treatment with TCA or JTE-013 in *kras*+ and WT zebrafish. Livers were recognized by GFP fluorescence in *kras*+ larvae and outlined in WT larvae. (**B**) 2D measurements of liver size in different groups. (**C**) Representative images of liver sections stained for Alcam in different groups. (**D**) Quantification of Alcam stained cholangiocytes. (**E**) Co-immunostaining of Alcam and pERK. Alexa Fluor 546 secondary antibody staining was used for Alcam and pERK detection. pERK stained signal is nucleus-localized as exampled in insets and indicated by arrowheads while Alcam staining is more on cell membrane as exampled and indicated by arrows in insets. (**F**). Quantification of pERK stained cholangiocytes. N = 10 each group. Scale bar: 200 μm (**A**) and 20 μm (**C**,**E**): Statistical significance: *P˂0.05.

To analyze the role of S1pr2 during hepatocarcinogenesis, both an agonist, taurocholate (TCA), and an antagonist, JTE-013, were used^42^. *kras* + and WT larvae were treated with TCA or JTE-013 in conjunction with Dox from 3 to 8 dpf. TCA led to a further increase in liver size compared to the group treated with Dox alone (Fig. 2A,B). JTE-013 had an opposite effect, as the liver size became significantly smaller than that in the Dox alone group (Fig. 2A,B). To confirm the link between S1pr2 and cholangiocytes, Alcam staining was used to determine cholangiocyte density in the same treatment groups. As shown in Fig. 2C and quantified in Fig. 2D, cholangiocyte density in *kras* + larvae was significantly increased in the TCA/Dox group compared to that in the control (Dox alone) group, while the JTE-013/Dox group showed a lower cholangiocyte density than the control group. In comparison, there was no significant change of cholangiocyte density in WT larvae by the two chemicals (Fig. 2A,B). In addition, co-staining for Alcam and pERK (a known downstream marker for S1pr2 activation in intrahepatic cholangiocarcinoma (ICC)^44^, indicating elevation and inhibition in pERK signal (nucleus-localized) in cholangiocytes by TCA and JTE-013 respectively (Fig. 2E,F).

**Figure 3:**
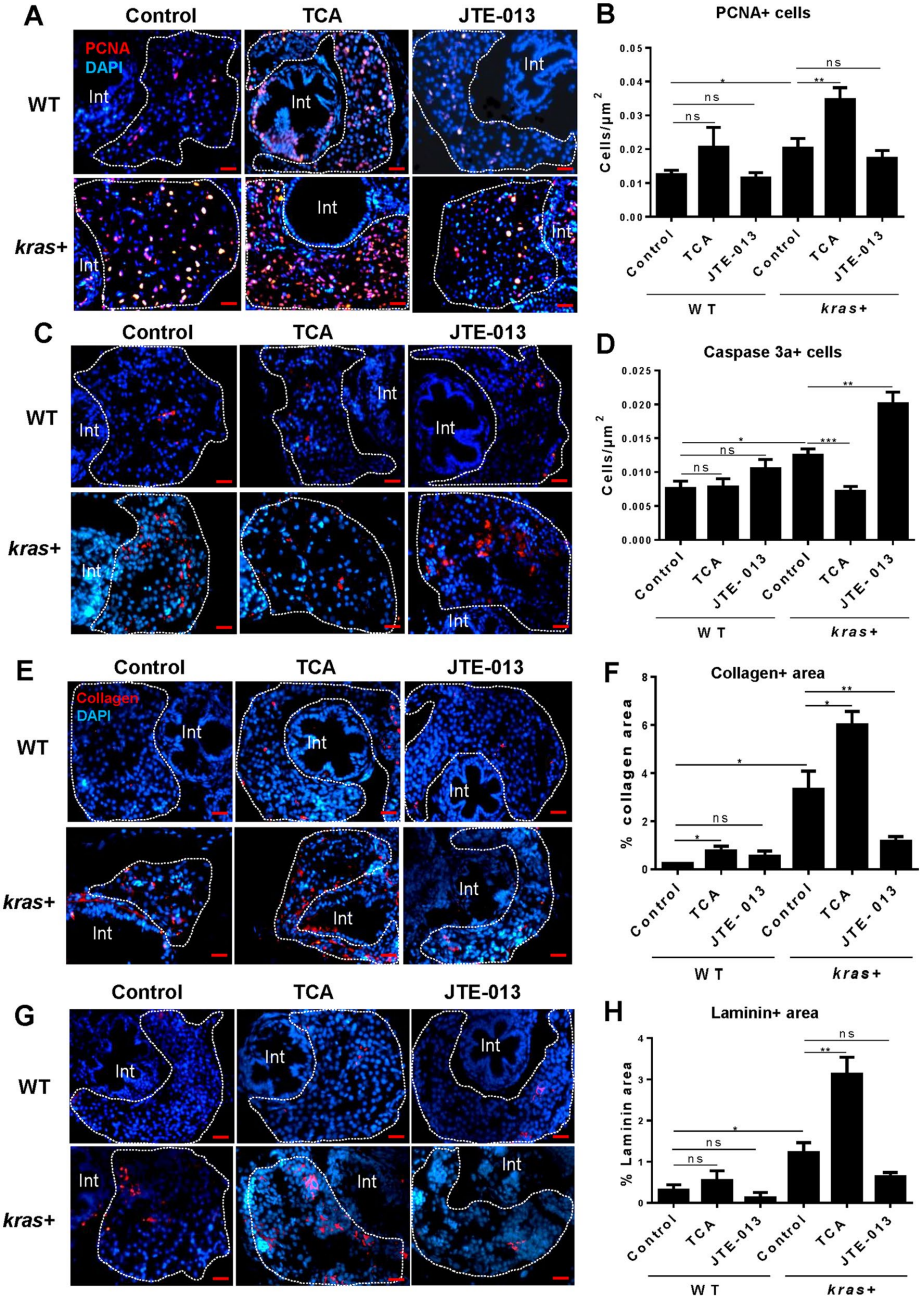
Effect of cholangiocyte activation and inhibition on hepatocyte proliferation, apoptosis and fibrosis. 3-dpf *kras* + and WT zebrafish larvae were treated with either TCA or JTE-013 along with 20 µg/mL Dox till 8 dpf. Samples were collected for immunohistochemistry. *kras*+ and WT liver sections were incubated with primary antibodies for PCNA, Caspase 3a, Collagen or Laminin and then stained with Alexa Fluor 546 conjugated secondary antibody. All liver sections were counter-stained with DAPI. (**A**,**C**, **E**,**G**) Representative images of staining for PCNA (**A**), Caspase 3a (**C**), Collagen (**E**) and Laminin (**G**) of *kras*+ and WT zebrafish larvae in different groups. (**B**,**D**, **F**,**H**) Quantification of staining signals. For PCNA and Caspase 3a staining, number of stained cells were quantified. For Collagen and Laminin stainings, stained areas were quantified. N = 10 each group. Scale bar: 20 μm. Statistical significance: *P˂0.05.

To further characterize the effects of S1pr2 on hepatocarcinogenesis, molecular markers for proliferation, apoptosis and fibrosis were examined by immunohistochemical staining after TCA and JTE-013 treatments. Cell proliferation, as determined by PCNA staining, were further increased by TCA and reduced, though not significantly, by JTE-013 in the *kras* + livers (Fig. 3A,B). Based on Caspase 3a staining, cell apoptosis was reduced by TCA and greatly enhanced by JTE-013 in the *kras* + livers (Fig. 3C,D). Consistently by Laminin and Collagen staining (Fig. 3E-H), these fibrosis markers showed great increases by TCA and decreases by JTE-013 in the *kras* + livers. Thus, the agonist TCA enhanced liver cell proliferation and fibrosis and reduced apoptosis while the antagonist JTE-013 showed exactly the opposite effects, indicating the general positive role of S1pr2 in liver cell proliferation and fibrosis and a negative role in liver cell apoptosis.

### Crosstalk between oncogenic hepatocytes and cholangiocytes

It is well known that triglyceride accumulation in the liver, or non-alcoholic steatohepatitis (NASH), increases the risk for development of HCC and ICC^47^. We hypothesized that in the *kras*^*V12*^ transgenic model, oncogenic hepatocytes develop NASH by accumulating triglycerides, which are converted to bile acids by Cholesterol 7 alpha-hydroxylase (Cyp7a1), the main enzyme in the classical pathway of bile acid synthesis^48^. Bile acids may promote cholangiocyte proliferation through S1pr2 activation. To test the hypothesis, we examined triglyceride accumulation in the liver upon *kras* induction in hepatocytes. As shown in Supplementary Fig. S2A,B, Oil red O staining of liver triglycerides showed a significant increase in triglyceride accumulation in the *kras* + group than those in the WT group at all time points from 8 to 96 h following the induction of oncogenic *kras*^*V12*^ expression. This finding confirmed that *kras*^*V12*^ induced hepatocarcinogenesis is accompanied with hepatic steatosis. Consistent with this, *cyp7a1* mRNA was 22-folds higher in *kras* + hepatocytes than WT hepatocytes (Supplementary Fig. S2C).

**Figure 4:**
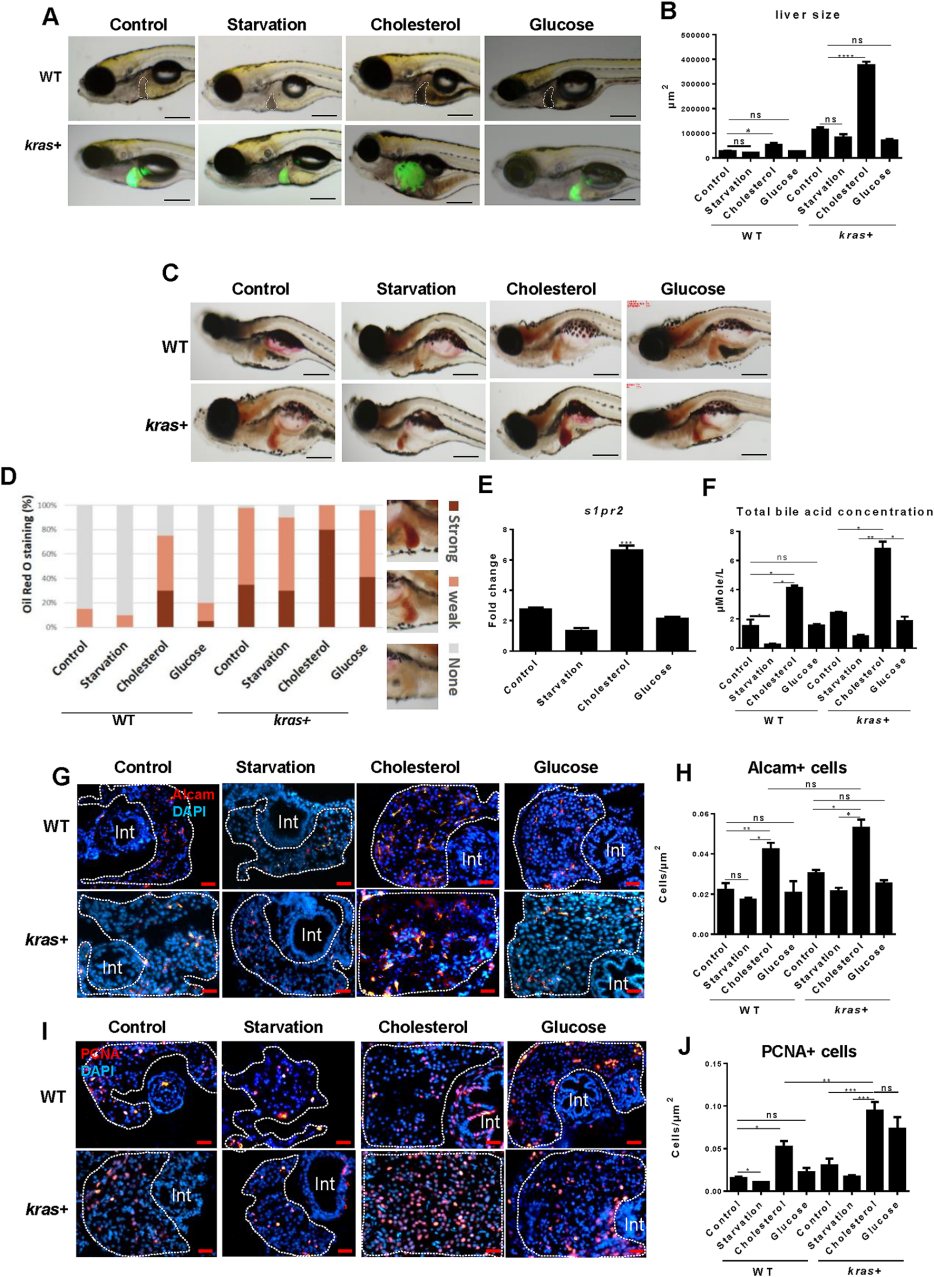
Effects of differential feeding on liver tumorigenesis. 7-dpf *kras*+ and WT zebrafish larvae were divided into four groups: control group (normal diet), starvation group (no diet), cholesterol group (10% cholesterol supplement) and glucose group (10% glucose supplement). These larvae were fed in these different regimes till 12 dpf, 20 µg/mL Dox treatment was applied to all groups till 12 dpf for various analyses. (**A**) Representative images of *kras*+ and WT larvae (12 dpf) to shows liver size in different feeding groups. Livers were recognized by GFP fluorescence in *kras*+ larvae and outlined in WT larvae. (**B**) 2D measurements of liver size in different groups. (**C**) Representative images of Oil red O staining of the *kras*+ and WT larvae in different feeding groups. (**D**) Quantification of Oil red O staining intensity in different groups. Examples of “Strong”, “Weak” and “None” staining are shown on the right of the histogram. (**E**) Fold change of *s1pr2* mRNA expression in *kras*+ larvae over that of WT larvae in the same feeding regime. (**F**) Concentrations of total bile acid concentration in zebrafish larvae under different feeding conditions. Total bile acids were determined as µmol/L at 12 dpf. (**G**–**J**) Representative images of Alcam (**G**) and PCNA (**I**) staining and their quantification (**H**,**J**) in different feeding groups. N = 10 per group. Scale bar: 200 μm (**A**,**C**) and 20 μm (**G**,**I**). Statistical significance: *P˂0.05.

To further test our hypothesis, a differential feeding experiment was carried out with 10% cholesterol (to induce NASH in the liver), 10% glucose, normal feed or starvation from 5 to 12 dpf (see Method). In both *kras* + and WT groups, the liver size was significantly larger in the cholesterol feeding group than those in the other three groups (Fig. 4A,B). Hepatic triglyceride accumulation was also higher in the cholesterol group than in the other three groups (Fig. 4C,D). Thus, *kras* + larvae may have a priority for either de novo synthesis or storage of fatty acids to support tumor growth and proliferation.

To confirm the link between hepatic triglyceride accumulation and S1pr2, *s1pr2* mRNA was measured in different feeding groups. Indeed, *s1pr2* mRNA was significantly higher in the cholesterol group than in the other groups (Fig. 4E), indicating that NASH induction may also activate *s1pr2* expression. Furthermore, when total bile acid was determined, we found that its concentration was significantly higher in the cholesterol group than those in the normal feeding group in both *kras* + and WT larvae, confirming that the increase in hepatic triglycerides is associated with an increase in the total bile acid content (Fig. 4F). Finally, there was an increase in both cholangiocyte density (Fig. 4G,H) and PCNA + cells (Fig. 4I,J) in the cholesterol group than that in the normal feeding group in both *kras* + and WT larvae, thus providing an additional clue that fatty liver has an impact on cholangiocyte proliferation probably through bile acid activation of S1pr2. The activated cholangiocytes in turn influence liver cell proliferation and carcinogenesis.

### Molecular feedback mechanism of cholangiocytes to oncogenic hepatocytes

**Figure 5:**
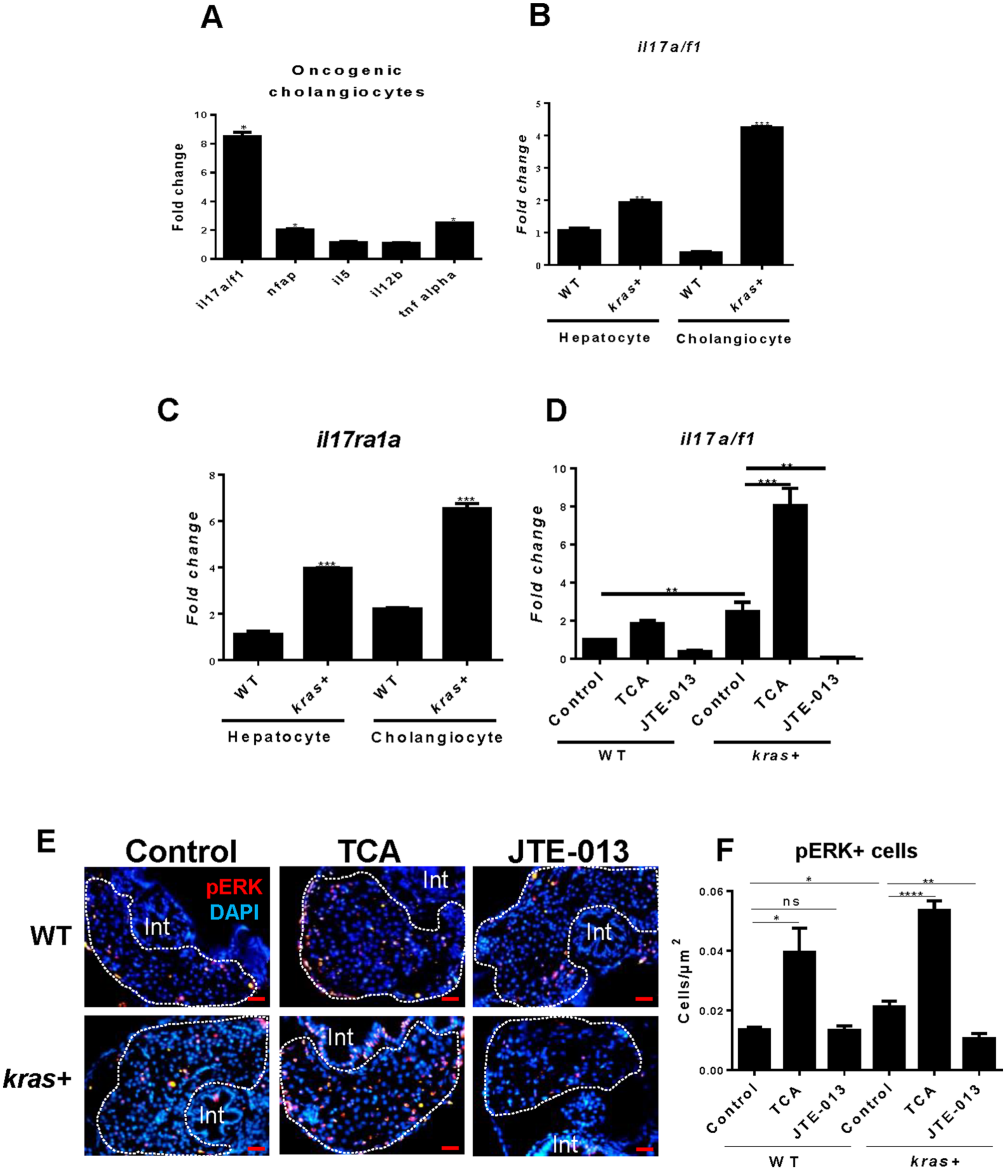
Expression of selected cytokine mRNAs in hepatocytes and cholangiocytes of the liver in adult zebrafish. (**A**) Expression of selected cytokine mRNAs in cholangiocytes upon *kras* induction in hepatocytes. (**B**, **C**) Expression of *il17a/f1* (**B**) and *il17ra1a* mRNAs in cholangiocytes and hepatocytes in WT and *kras*+ larvae following Dox induction from 3 to 8 dpf. (**D**) Expression of *il17a/f1* mRNA in *kras* + and WT larvae treated with TCA and JTE-013 together with Dox from 3 to 8 dpf. In (**A**), gene expression values in the *kras* groups were normalized to their WT counterparts. In (**B**–**D**), all values were normalized to WT hepatocyte value. (**E**, **F**) Representative images of il17a/f1 downstream maker p-ERK staining (**E**) and quantification of pERK stained cells (**F**) in *kras*+ and WT zebrafish larvae treated with TCA and JTE-013 from 3 to 8 dpf. N = 10 per group. Scale bars, 20 μm Statistical significance: *P˂0.05.

Cholangiocytes may influence hepatocyte carcinogenesis by secreting pro-inflammatory cytokines. To investigate this possibility, cholangiocytes and hepatocytes were isolated by FACS for RNA extraction. Selected cytokine genes were analyzed for their expression in cholangiocytes by RT-qPCR. We found that *il17a/f1 (interleukin 17a/f1),* a proinflammatory cytokine gene, was about four fold up-regulated in *kras* + cholangiocytes compared to that in WT cholangiocytes (Fig. 5A). In comparison, expression of other tested cytokine genes including *tnfα* (*tumor necrosis factor alpha*), *nfap (nuclear factor activating protein*) *il5 (interleukin 5*) and *il12b (interleukin 12b*) showed much less increase in *kras* + cholangiocytes. Furthermore, the induction of *il17a/f1* in *kras* + cholangiocytes was much higher than that in *kras* + hepatocytes (Fig. 5B). Interestingly, the induction of Il17a/f1 receptor (*il17ra1a*) mRNA expression appeared to be high in both hepatocytes (3.9 fold) and cholangiocytes (6.5 fold) in *kras* + fish (Fig. 5C). Thus, Il17a/f1 secreted from cholangiocytes may exert its effect on hepatocytes upon oncogenic *kras* induction in hepatocytes. Finally, to link the changes with S1pr2, we analyzed the expression of *il17a/f1* in zebrafish larvae after treating them with S1pr2 agonist (TAC) or antagonist (JTE-013) to activate or suppress cholangiocytes. We indeed noticed up- and down-regulation of *il17a/f1* expression by TAC and JTE-013 respectively (Fig. 5D). This observation support that the tumorigenic effect of cholangiocytes on hepatocytes might be via *il17a/f1*.

To further investigate the stimulating role of cholangiocytes upon oncogenic hepatocytes via the IL17a/f1 pathway, we analyzed the expression pattern of downstream markers of Il17a/f1 receptor by immunostaining of specific downstream marker pERK. As presented in Fig. 5E and quantified in Fig. 5F, pERK immunostaining signal was significantly expressed in the liver of *kras* + zebrafish larvae upon TCA activation of cholangiocytes. In contrast, inhibiting cholangiocytes via JTE-013, led to subsequent decrease in pERK than the control group.

### Determent of HCC progression by Il17a/f1 morpholino knockdown

IL17A family has been shown to be involved in several types of cancers^49–51^. To validate the effect of *il17a/f1* on tumor development and infiltration of immune cells (neutrophils and macrophages) to the liver, *il17a/f1* was specifically knocked down via two different morpholinos: one targeted at *il17a/f1* translation start site (Trs_Mo) and the other at an *il17a/f1* splicing site (Spl_Mo). A control morpholino (Ctr_Mo) targeting at human beta-globin gene was also used. As shown in Supplementary Fig. S3, both Trs_Mo and Spl_Mo reduced liver size significantly in the *kras* + larvae compared to uninjected or Ctr_Mo injected groups. Three independent microinjection experiments were carried out for these mopholinos and all experiments showed consistent results.

**Figure 6:**
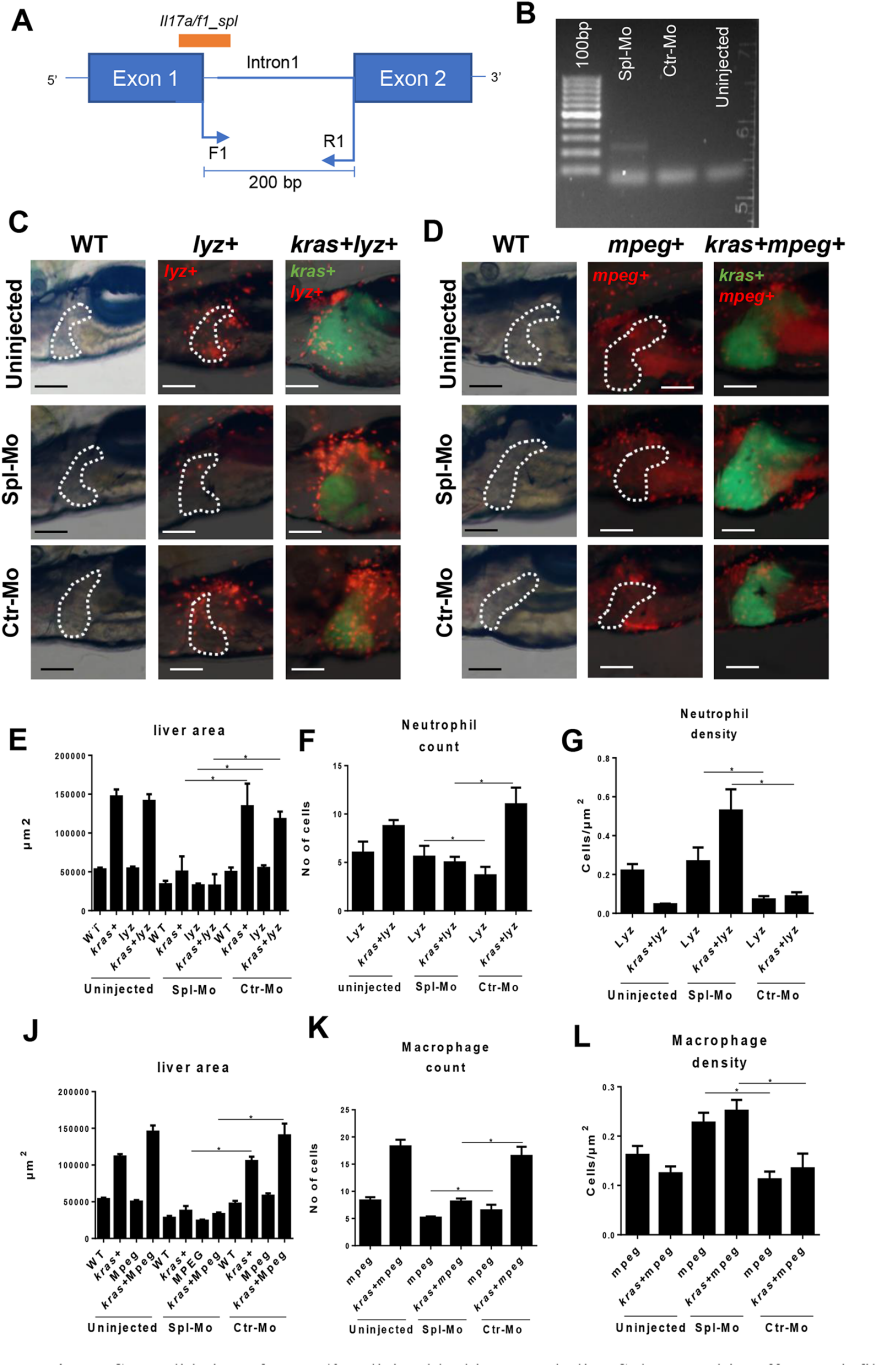
Validation of *iL17a/f1* splicing blocking morpholino Spl-Mo and its effect on infiltration of neutrophils and macrophages in the liver. (**A**) Diagram of *iL17a/f1* gene for the targeted sites of Spl-Mo and PCR primers. A 200-bp fragment is expected when Spl-Mo blocks the splicing. (**B**) Agarose gel electrophoresis of RT-PCR products after introduction of Spl-Mo. Mopholinos were introduced into zebrafish embryos at one cell stage and RNA was isolated at 6 hpf for RT-PCR analysis. (**C**, **D**) Representative images of liver-infiltrated neutrophils (**C**) and macrophages (**D**) after *il17a/f1* knockdown by Spl-Mo. *Lyz*+ and *mpeg*+ transgenic zebrafish were used for marking neutrophils (dsRed expression) and macrophages (mCherry expression) respectively and these transgenic fish were compounded with *kras*+ zebrafish for investigation of liver-infiltrated immune cells. Livers are outlined for non-*kras*+ samples. (**E**–**G**) Quantification of liver size (**E**), number (**F**) and density (**G**) of liver-infiltrated neutrophils. (**J**–**L**) Quantification of liver size (**J**), number (**K**) and density (**L**) of liver-infiltrated macrophages. N = 10 per group. Scale bars: 100 μm. Statistical significance: *P˂0.05.

To validate the splicing blocking morpholino Spl_Mo, which targeted the first intron–exon junction, a pair of PCR primers flanking the first intron were used to monitor an anticipated 200-bp fragment of the splice blocked target. After gel electrophoresis of RT-PCR products from embryos injected or uninjected with morpolinos, a band of 200 bp size only appeared in the embryos injected with Spl_Mo morpholino but not in the control Ctr_MO and uninjected group (Fig. 6A,B).

To further validate the effect of knockdown of *il17a/f1*, two immune cell reporter transgenic lines were employed, *lyz* + for dsRed + neutrophils and *mpeg* + for mCherry + macrophages. *spl–il17a/f1* morpholino was injected into *kras* + *, kras* + */lyz* + *, lyz* + *, kras* + */mpeg* + *, mpeg* + and WT embryos at one-cell stage and injected embryos were analyzed for liver size, dsRed + neutrophil counts and density, mCherry + macrophage count and density within the liver. As shown in Fig. 6C,E, by 6 dpf, there was an overall decrease of liver size in spl-MO injected larvae, compared to those in *kras* + larvae injected with Ctr_MO and uninjected group. Furthermore, we noticed that *il17a/f1* knockdown led to decreases of both number and density of infiltrated neutrophils to the liver (Fig. 6F,G). Similarly, decreases of liver size, number and density of infiltrated macrophages to the liver by *il17a/f1* knockdown were also observed (Fig. 6D,J–L). Collectively, our data have shown that cholangiocytes could accelerate HCC progression through Il17a/f1 cytokine which could be a potential target for cancer therapy.

## Discussion

Liver is the largest internal organ, consisting of 70% hepatocytes and 15% cholangiocytes. Hence cholangiocytes represent the second largest population of cellular entity of the liver and they are important to maintain liver homeostasis after hepatocyte loss and inflammation^52–54^. In our study, we used an established transgenic zebrafish model to overexpress *kras*^*V12*^ oncogene in hepatocytes to initiate hepatocarcinogenesis and investigated the interaction between oncogenic hepatocytes and neighboured cholangiocytes. Our data showed that there was a rapid and consistent increase of cholangiocytes from 8 h post Dox treatment. Cholangiocyte number and density continued to increase over the 96 h of Dox treatment compared to hepatocytes in WT larvae. Our data also showed a firm correlation between cholangiocyte density and liver size increase after *kras*^*V12*^ activation in hepatocytes. Further pharmacological experiments were performed to confirm the bond between the two main types of liver cells, hepatocytes and cholangiocytes.

The pharmacological amenability of cholangiocytes to different drugs depend primarily on receptor of choice on cholangiocytes surface. Under different physiological conditions such as inflammation and cholestasis, cholangiocytes express specific receptors to promote their proliferation, such as S1pr2^44^, estrogenic receptors^8^, muscarinic receptor^55^ and secretin receptor^9^. Among those receptors, S1pr2 showed the most dramatic increase in cholangiocytes after *kras*^*V12*^ induction in hepatocytes. Also, the same receptor has been reported to be activated in human cholangiocarcinoma cell lines^42–44^ as well as after bile duct ligation in mouse models^56^. By using specific agonist (TCA) or antagonist (JTE-013) to S1pr2, we demonstrated that liver tumorigenesis became further enhanced or deterred respectively. Upon cholangiocyte activation, hepatocytes proliferation and fibrosis were found to be increased while apoptosis was decreased; these are signs of enhanced HCC progression. Consistent with the above observations, it has been reported that activated cholangiocytes can accelerate liver fibrosis by different mechanisms, firstly by secreting profibrotic factors such as connective tissue growth factor (CTGF). Secondly laminin synthesis can occur in cholangiocytes as in rat cholestatic models. Finally, cholangiocytes can induce fibrosis directly in hepatocytes by promoting epithelial mesenchymal transition (EMT) or indirectly by promoting other hepatic cells^8,9,11^.

Hepatocytes and cholangiocytes interact reciprocally in the liver under different physiological conditions^57–59^. One of the earliest studies to dissect the interaction between hepatocytes and cholangiocytes was performed in a rat model of hepatocarcinogenesis induced by ethionin, Novikoff et al. showed that multiple intercellular junctions connect hepatocytes, cholangiocytes and bile canalicular structure. These junctions are responsible for the intercellular transport of specific soluble factors that can cause changes in the histology and ultrastructure of both cell types^39^. Here we attempted to go further to identify the molecular crosstalk between the two types of cells in our *kras*^*V12*^ zebrafish model. Upon *kras*^*V12*^ induction in adult hepatocytes for 7 days, a dramatic increase in the expression of *cyp7a1* mRNA was observed in oncogenic hepatocytes compared to WT hepatocytes. *Cyp7a1* is the main gene in the classical pathway of bile synthesis from cholesterol precursor. Recent reports indicate that the bile acids can directly activate *s1pr2*^60^. Consistent with this, we also found that the concentration of total bile acids was also increased in kras + larvae, especially when they were fed with high cholesterol diet (Fig. 4). The increase of total bile acids correlates with the increase of the size of oncogenic *kras* + livers. Thus, there should be a connection of the bile acids in activating cholangiocytes during liver tumorigenesis.

Our data also showed that *il17a/f1* was up-regulated in cholangiocytes upon oncogenic activation of hepatocytes while its receptor, *il17ra1a*, was up-regulated in hepatocytes; thus, the effect of cholangiocytes on hepatocytes is likely via Il17a/f1 pathway. Consistent with this, staining of Il17a/f1 downstream marker pERK showed a positive correlation with cholangiocyte activation by its agonist TCA (Fig. 2E,F). To further validate the role of Il17a/f1 released from cholangiocytes on promoting liver tumorigenesis, we performed *il17a/f1* morpholino knockdown experiments on *kras*_+_ zebrafish larvae. Indeed, knockdown of *il17a/f1* caused a deferment of liver tumorigenesis as judged by decreased liver size (Fig. 6). Overall, the crosstalk between hepatocytes and cholangiocytes was mostly based on the correlated expression of *il17a/f1* cytokine gene and its receptor gene (*il17ra1a*) and a preliminary *il17a/f1* experiments. In future, further works to specifically localize Il17a/f1 and Il17ra1a in cholangiocyte and hepatocytes and characterization of their function are needed. However, consistent with our current observations, previous reports also indicated elevated expression of IL17 in human HCC samples^51^ and its role in enhancing HCC inflammatory environment^61–65^. Previously, we also confirmed that inflammatory immune cells play a prominent role during HCC progression^35^.

**Figure 7:**
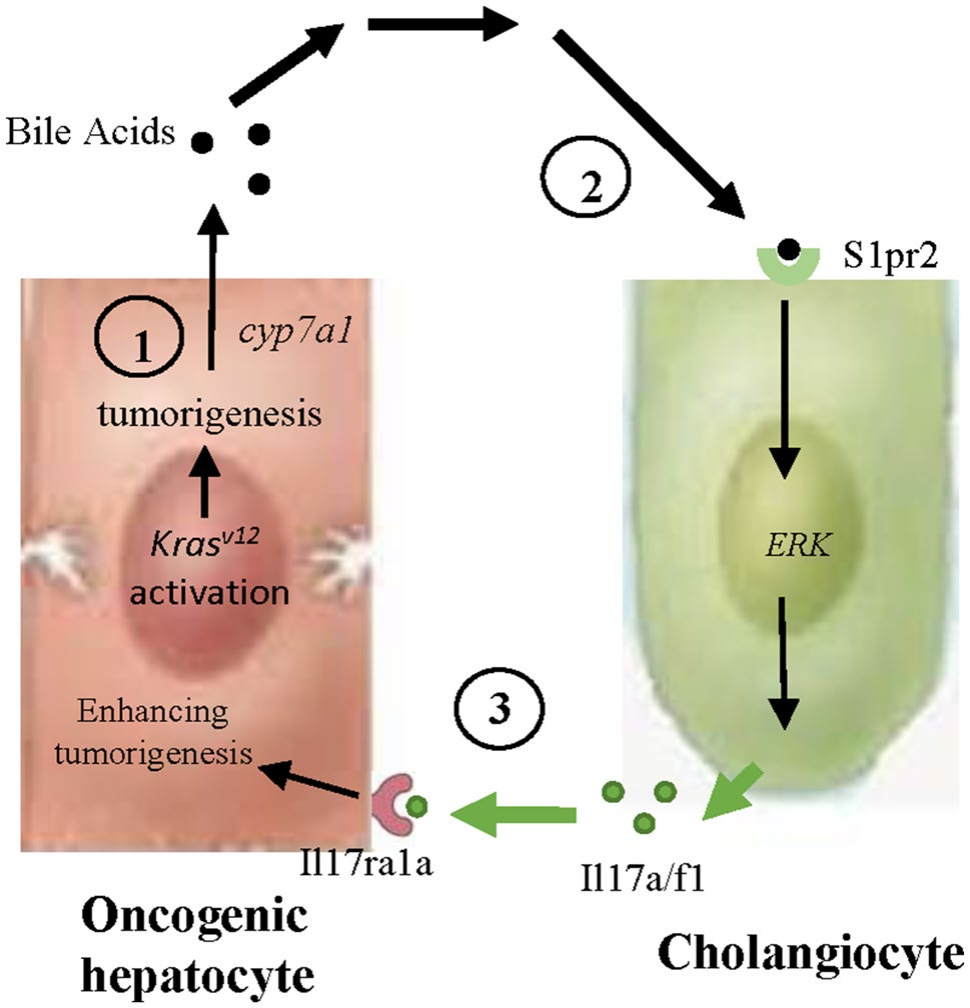
Proposed model for interaction between oncogenic hepatocytes and cholangiocytes in the *kras* + transgenic zebrafish model. First, oncogenic hepatocytes after *kras*^*v12*^ activation promote accumulation of triglycerides and elevate secretion of bile acids. Second, bile acids induce cholangiocyte proliferation through S1pr2, which, upon activation, induces pERK and secretion of Il17a/f1 cytokine. Third, Il17a/f1 binds to its receptor Il17ra1a on hepatocytes and promotes tumorigenesis through an inflammation pathway.

In summary, our data suggested that cholangiocytes play an important role in promoting HCC development through an inflammatory loop. While hepatocytes increase bile acids synthesis and lipogenesis to satisfy its demanding need for energy, cholangiocytes respond positively to hepatic bile acids and induce pro-inflammatory environment through Il17a*/f1* secretion and other cytokines. This in turn accumulates more inducing signals for hepatic carcinogenesis. A proposed model for the interaction between oncogenic hepatocytes and cholangiocytes upon *kras*^*V12*^ induction in hepatocytes is presented in Fig. 7. In future, it will be interesting to investigate whether the stimulating role of Il17a/f1 is only specific to *kras*-induced cancer or universally to most other cancers. Understanding of this should provide valuable information for development of effective therapeutic approaches.

## Materials and methods

### Zebrafish husbandry

Zebrafish were maintained in compliance with the guideline of Institutional Animal Care and Use Committee (IACUC) of the National University of Singapore (NUS) and the protocol was approved by IACUC of NUS (Protocol Number: 096/12). Transgenic zebrafish models used in the current study include *Tg(fabp10:rtTA2s-M2; TRE2:EGFP-krasG12V)* (*zg32Tg*) in a tetracycline-controlled transcription activation (Tet-On) system for inducible expression of oncogenic *kras*^*V12*^ in hepatocytes^25^, *Tg*(*lyz:DsRed2*) (nz50Tg) with DsRed-labeled neutrophils under the lysozyme C (*lyz*) promoter^66^, Tg(*mpeg1:mCherry*) (*gl22Tg*) with mCherry-labeled macrophages under the *mpeg1* promoter^67^, and *Tg(fabp10a:dsRed;ela3l:GFP) (zg15Tg)*^68^. These transgenic lines were referred to as *kras*+, *lyz*+, *mpeg*+, and *fabp10a* + respectively, in this report.

### Chemical treatment

All drug/chemical treatments were conducted in 3-dpf (day postfertilization) larvae for 5 days. The reagents used in this study include; doxycycline (Dox) (D9891; Sigma-Aldrich, St. Louis, MO), JTE-013 (J4080, sigma), TCA (86,339, Sigma) and cholesterol (Sigma). Concentrations used were 20 µg/ml Dox, 10 µM JTE-013, 100 µM TCA and 10% cholesterol.

### Induction of zebrafish NASH by cholesterol feeding

Supplement-enriched diets were prepared as previously described^29^. Briefly, cholesterol (Sigma) was dissolved in dimethyl sulfoxide (DMSO) to make a 10% solution, of which 400 μl was added to 0.5 g of standard zebrafish larval food (dried algae). The diet was left to dry overnight, grounded to powder form and provided to treated larvae on daily basis. The same was followed for 10% glucose enriched diet. Finally, starvation group was deprived from feeding throughout the experimental period.

### Oil R O staining

Zebrafish larvae were fixed in 4% paraformaldehyde (PFA) (Sigma) in phosphate-buffered saline (PBS) at 4 °C and incubated in 60% 2-propanol for 10 min, followed by whole mount staining with freshly prepared/filtered 0.3% Oil Red O in 60% 2-propanol.

### Morpholino knockdown of *il17a/f1*

Two morpholino oligonucleotides targeting an RNA splice site (Spl-Mo, GTTCACTTCAGCTATACTCACCATA) and the translation site (Trs-Mo, CGGAGGTTTAACGCTGATGACAT) of *il17a/f1* were designed and synthesized by Gene Tools (Philomath, OR). A standard control morpholino (Ctr_Mo, 5′-CCTCTTACCTCAGTTACAATTTATA-3′) targeting a human beta-globin intron (Gene Tools, Philomath, OR) was also used as a negative control. Aliquots of morpholino (1 mM) and 1% (wt/vol) phenol red in Danieau solution were injected into embryos at the 1-cell stage. Dox was added to all larvae from 3 to 6 dpf.

To further validate the effect of splicing morpholino, RNA was isolated from 100 to 150 5-dpf larvae that were microinjected with Sp-Mo. A forward primer (ATGTCATCAGCGTTAAACCTCC) and reverse primer (ATGTAAGTCCATGGAGAGATGG) flanking the first intron were used to check for the presence of a 200-bp fragment from the first intron (Supplementary Fig. S3A).

### Determination of total bile acids

Concentration of total bile acids was determined in 10-dpf larvae by using a total bile acid quantification kit (Crystal Chem, 80470) according to manufacture protocol.

### Photography and image analyses

Zebrafish larvae after each treatment were collected, anesthetized immediately in 0.08% Tricaine (E10521; Sigma) and immobilized by using 3% methylcellulose (M0521; Sigma) before proceeding to imaging. Zebrafish larvae were photographed individually with an Olympus microscope (Olympus, Tokyo, Japan).

### Immunostaining and cytological analyses

Following the end of treatment, zebrafish Larvae were fixed in 4% PFA dissolved in PBS, embedded in bacterial-agar, and cryo-sectioned at 8-μm thickness using a cryotome. This was followed by immunohistochemical staining. Most of the primary antibodies used were derived from rabbits, including anti-proliferating cell nuclear antigen (PCNA) (FL-261, Santa Cruiz), anti–caspase 3 (Abcam), anti–collagen I (ab23730; Abcam) and anti-laminin (L9393, Sigma). Anti-Alcam (ANZN-8, ZIRC, USA) was derived from the mouse. Anti-rabbit or anti-mouse secondary antibodies were purchased from Thermo Fisher Scientific.

### Isolation of hepatocytes, neutrophils, macrophages and cholangiocytes by fluorescence activated cell sorting

*Fabp10*+, *lyz*+, and *mpeg*+ transgenic zebrafish lines in wild-type and *kras*+ background were used for fluorescence-activated cell sorting (FACS) isolation of hepatocytes, neutrophils, and macrophages respectively. 7–10 adult livers were pooled and dissociated into single cells in the presence of 0.05% trypsin (T1426; Sigma) by using a 40-μm mesh (352340; Fisher Scientific, Pittsburgh, PA) as previously indicated^69^. Hepatocytes (*fabp10*+) were isolated based on dsRed expression, neutrophils (*lyz*+) were isolated based on DsRed expression, and macrophages (*mpeg*+) were isolated based on mCherry expression. For isolation of cholangiocytes, Alcam antibody was used as an accepted cell surface marker for cholangiocytes together with the Alexa Fluor secondary antibody (Thermo Scientific).

### RNA extraction, complementary DNA amplification, and reverse-transcription quantitative polymerase chain reaction (RT-qPCR)

Total RNA was extracted from FACS isolated cells by using the RNeasy mini kit (74104; Qiagen, Singapore). Complementary DNA (cDNA) was synthesized and amplified by using QuantiTect Whole Transcriptome Kit (207043; Qiagen). Then the amplified cDNA was used as a template for quantitative polymerase chain reaction (qPCR) with LightCycler 480 SYBR green master mix (Roche, Singapore). cDNAs were subjected to 40 cycles with the following parameters (95 °C, 20 s; 65 °C, 15 s; and 72 °C, 30 s).

### Statistical analysis

Statistical significance between every two groups was evaluated by two-tailed unpaired Student t-test using GraphPad Prism version 7.00. Analyzed data are presented as mean values ± standard error of mean (SEM).

## Supplementary Information

Supplementary Information.
