# Author Correction: Stimulation of hepatocarcinogenesis by activated cholangiocytes via Il17a/f1 pathway in *kras* transgenic zebrafish model

**DOI:** 10.1038/s41598-021-92144-9

**Published:** 2021-06-18

**Authors:** Mohamed Helal, Chuan Yan, Zhiyuan Gong

**Affiliations:** 1 Department of Biological Sciences, National University of Singapore, Singapore, Singapore; 2 Marine Pollution Lab, Marine Environment Division, National Institute of Oceanography and Fisheries, Alexandria, Egypt

Correction to: *Scientific Reports* https://doi.org/10.1038/s41598-020-80621-6, published online 14 January 2021

The original version of this Article contained an error in the spelling of the author name Zhiyuan Gong which was incorrectly given as Zhiuyan Gong. The original Article and accompanying Supplementary Information file have been corrected.

