# Supplementary material for: Stimulation of hepatocarcinogenesis by activated cholangiocytes via Il17a/f1 pathway in kras transgenic zebrafish model
Source: Sci Rep. 2021 Jan 14;11:1372. doi: 10.1038/s41598-020-80621-6 (PMC7809472; doi:10.1038/s41598-020-80621-6)
Supplement: Supplementary file 1 — Supplementary Information. [file 41598_2020_80621_MOESM1_ESM.pdf]

Supplementary Information

Stimulation of hepatocarcinogenesis by activated cholangiocytes upon induction of oncogenic *kras* expression in transgenic zebrafish

Mohamed Helal[1,2], Chuan Yan[1], Zhiyuan Gong[1,*]

[1] Department of Biological Sciences, National University of Singapore, Singapore

[2] Marine pollution Lab, Marine Environment Division, National Institute of Oceanography and Fisheries, Alexandria, Egypt.

[*] Corresponding author, Dr. Zhiyuan Gong, Department of biological Sciences, National University of Singapore, Singapore. Email: dbsgzy@nus.edu.sg; phone: (65)-65162860; fax: (65)-67792486.

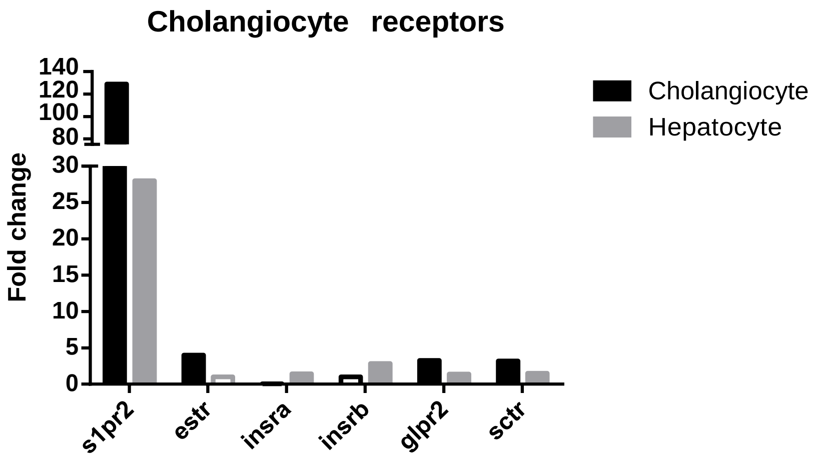

Supplementary Fig. S1. Expression of various cholangiocyte receptor mRNAs in zebrafish hepatocytes and cholangiocytes. Hepatocytes and cholangiocytes were isolated by FACS based on GFP and RFP expression, respectively, from adult *fabp10+* and *kras+* transgenic zebrafish. and mRNA expression was determined by RT-qPCR. All values were normalized to *kras-* counterparts and expressed as fold change.

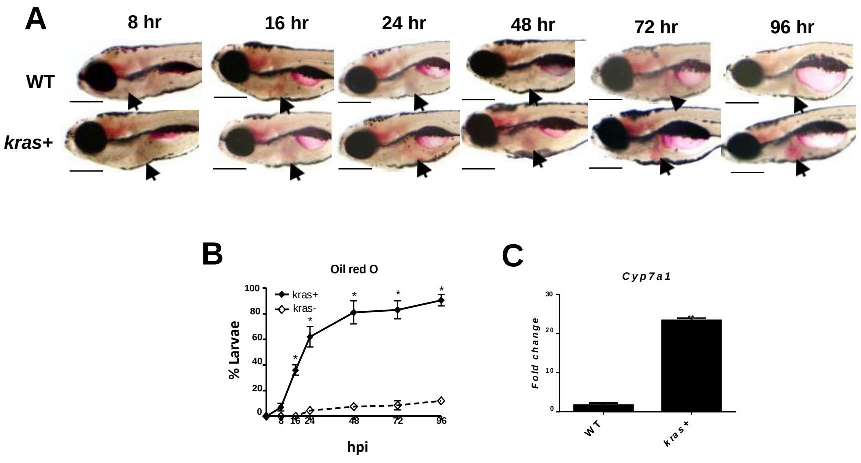

Supplementary Figure S2. Triglyceride accumulation during liver tumorigenesis. 3-dpf *kras*+ and WT zebrafish larvae were treated with 20 µg/mL Dox till 12 dpf. Samples were collected, fixed in 4% paraformaldehyde and used for Oil red O staining. (A) Representative images of Oil red O stinging of *kras*+ and WT larvae at different time points as indicated. Arrow points to lipid staining in the liver. (B) Quantification of Oil O Red in *kras*+ and WT larvae. Accumulation of triglycerides becomes obvious and significant starting from 8 hours post-induction by Dox in the *kras*+ group. (C) Comparison of levels of *cyp7a1* mRNA expression in hepatocytes of WT and *kras*+ adult zebrafish following Dox induction. Scale bars: 200 µm.

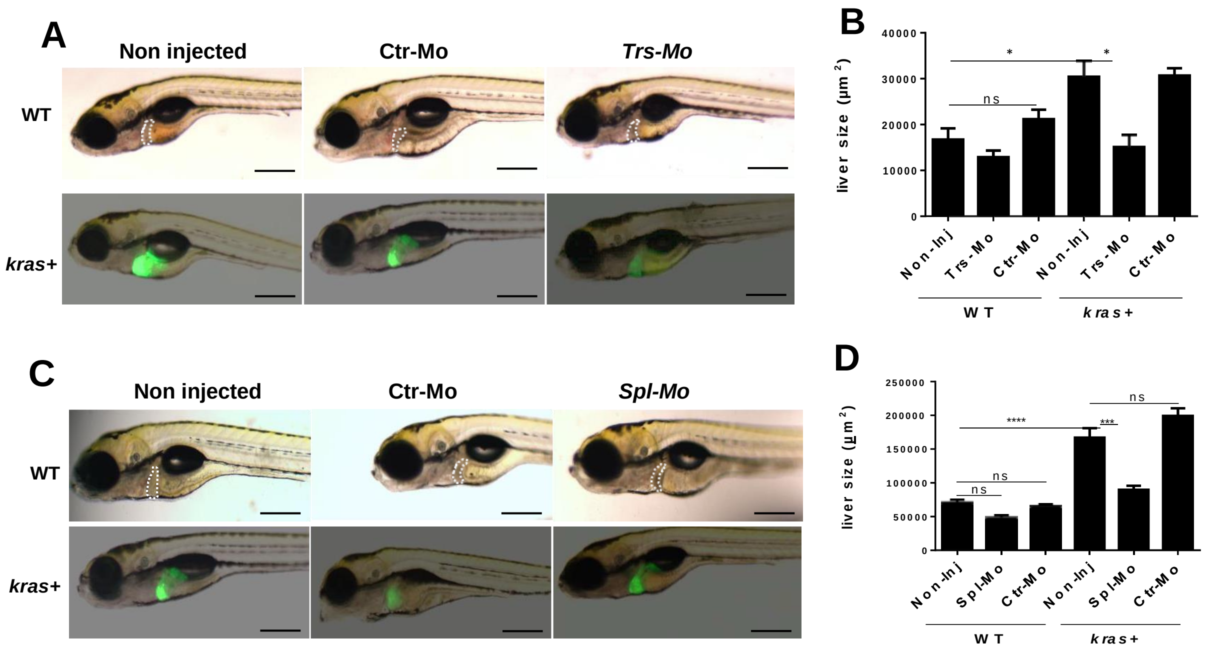

Supplementary Figure S3. Effect of morpholino knockdown of *il17a/f1* on liver size. Zebrafish embryos at one-cell stage were injected with *Trs-Mo* or *Spl-Mo* and liver size was examined at 6 dpf. Ctr-MO injected and non-injected embryos were used as controls. (A) Representative images of liver size after *il17a/f1* morpolino knockdown by translational-morpholino (Trs-Mo). Control morpholino (Ctr-Mo) and uninjected siblings of *kras+* and WT zebrafish larvae were included for comparison. Livers were recognized by GFP fluorescence in *kras+* larvae and outlined in WT larvae. (B) 2D liver size quantification in different groups. (C) Representative images of liver size after *il17a/f1* morpholino knockdown (Spl-Mo). (D) liver size quantification in different groups. N ≥15 each group. Scale bars, 50 µm. Statistical significance: $*P<0.05$.
